# Diffusion of Silver Diamine Fluoride Solution in Dentine: An In Vitro Study

**DOI:** 10.1016/j.identj.2024.06.002

**Published:** 2024-08-03

**Authors:** Ahmed Zaeneldin, Chun Hung Chu, Ollie Yiru Yu

**Affiliations:** Faculty of Dentistry, The University of Hong Kong, Hong Kong, S.A.R., China

**Keywords:** Silver diamine fluoride, SDF, Dental caries, Prevention, Dental pulp, Dentine

## Abstract

**Objectives:**

The aim of this research was to assess the diffusion dynamics of silver and fluoride ions after 38% silver diamine fluoride (SDF) solution application on dentine of varying thicknesses over 24 weeks.

**Methods:**

Bovine dentine discs of 5.5 mm in diameter were prepared and separated into 3 groups with thicknesses of 0.5 mm (group 1), 1.0 mm (group 2), and 1.5 mm (group 3). The diameter and number of dentinal tubules of discs were assessed. Each disc received a topical application of 0.05 mL 38% SDF solution. The deionised water in the tube was collected weekly for 24 weeks. The silver and the fluoride ion concentrations in the collected deionised water were determined. Generalised estimating equations was used to explore the potential effects of the key factors on the silver/fluoride diffusion.

**Results:**

The amount of silver and fluoride ion diffusion through dentine almost levelled off after 4 weeks and showed a decline trend over 24 weeks. The mean (SD) 24-week cumulative ion diffusion through dentine in groups 1, 2, and 3 was as follows: 20 (4) μg, 10 (2) μg, and 5 (1) μg for silver (*P* < .05) and 18 (2) μg, 13 (2) μg, and 7 (1) μg for fluoride (*P* < .05), respectively.

**Conclusions:**

Silver and fluoride ion diffusion through dentine showed a decline trend over 24 weeks. The diameter and the number of dentinal tubules on dentine with different thicknesses affects the ion diffusion dynamics. This study provides indications on the pattern of silver/fluoride ions diffusion through dentine to pulp after 38% SDF application. An increased amount of silver/fluoride diffuses through dentine into the pulp with decreased remaining dentine thickness.

## Introduction

Dental caries is the predominant dental disease impacting individuals across all age groups.^1^ The condition arises from biofilm accumulation on tooth surfaces, leading to acid production that can deteriorate the structural integrity of the enamel and dentine.^2^ Deep carious lesions represent an advanced stage of dental caries, manifesting the decayed cavity with proximity to the pulp. If these deep carious lesions are left untreated, acute and chronic pulpitis, pain, discomfort, vitality loss, tooth loss, abscesses, or potential systemic infections may result.^3^ The socioeconomic implications of untreated dental caries are significant and include increased health care costs, missed work or school days, and reduced overall quality of life.^4^

Silver diamine fluoride (SDF) has emerged as a noninvasive treatment option for dental caries. SDF application is straightforward and pain-free. SDF can be easily applied to the affected teeth using a brush or cotton applicator.^5^ Notably, SDF has been validated for its efficacy in preventing dental caries and arresting caries progression. Its effectiveness, particularly amongst children who might find traditional treatments challenging, has been well documented.^6^, ^7^, ^8^ Several systematic reviews provide robust evidence supporting the effectiveness of SDF application in arresting caries progression in primary teeth and in arresting existing and preventing new carious lesions on root surfaces.^9^, ^10^, ^11^ SDF is a clear solution containing a high concentration of silver and fluoride ions. It has an antimicrobial effect against cariogenic pathogens and remineralisation effects on carious lesions.^12^ The silver ions in SDF possess antimicrobial capabilities to inhibit the growth of cariogenic pathogens and deleterious metabolites that leads to caries progression, whereas the fluoride ions facilitate the remineralisation process.^13^

The fluoride and silver ions from SDF might diffuse through dentinal tubules and reach dental pulp after SDF treatment on caries lesions. In most clinical situations, SDF is applied onto the dentine of the cavity walls of the carious lesion. The direct application of SDF onto the cavity with dental pulp exposure is not recommended due to its high fluoride and silver ion concentration with an alkaline pH value of 10.^14^^,^^15^ In carious lesions without pulp exposure, the remaining dentine between the cavity floor and the dental pulp serves as an intermediary between the SDF and the pulp tissue.^16^ Dentine has a unique tubular structure with numerous microchannels, which allows ion transition and movement. A study by Luong et al^17^ reported that silver and fluoride ion diffusion was within the safety range after SDF and potassium iodide (KI) application on the external surface of the proximal wall with a fixed dentine thickness.

The thickness of dentine between the cavity floor and the dental pulp may play a key role in ion diffusion or leach rate through dentine.^18^ The thickness of remaining dentine indicates the distance of ion diffusion. Thinner dentine may indicate a high ion diffusion rate.^19^ In addition, the remaining dentine thickness affects the diameter and the number of dentinal tubules on the cavity floor of the lesion. According to the histologic structure of the teeth, when the cavity floor is closer to the dental pulp, a larger diameter and tubules might be observed.^20^ Therefore, the diameter and the number of dentinal tubules as well as the dentine thickness might be critical parameters the ion diffusion rate. However, evidence was limited in regards to ion diffusion rate in terms of reaching the dental pulp following SDF application.

Understanding the amount of fluoride and silver ions from SDF reaching the dental pulp through dentine of varying thicknesses is important to estimate the dental pulpal response to SDF. Therefore, the objectives this study are to assess the diffusion dynamics of silver and fluoride ions after 38% SDF application on dentine of varying thicknesses over 24 weeks and to explore the impact of the diameter and the number of dentinal tubules on dentine of varying thicknesses on ion diffusion dynamics. The null hypothesis is that there is no difference in the amount of silver and fluoride ions diffusing through the dentine, irrespective of the any of the previously mentioned parameters.

## Materials and methods

### Specimen preparation

Bovine incisors’ roots were cut using a low-speed custom-made diamond saw at the cementoenamel junction. The roots were cut vertically to obtain dentine specimens from the area around the cementoenamel junction and superficial to the dental pulp cavity. Specimens with cracks or any defects were excluded. A high-speed handpiece with a diamond straight stone #2878 (Komet) was used to shape the dentine specimens into circular discs 5.5 mm in diameter under copious water irrigation. Random allocation of specimens was carried out to divide them into 3 groups, each group consisting of 5 dentine discs. To simulate the remaining dentine between the cavity floor of caries lesions in the clinical scenario, the dentine tissue close to the pulpal side was preserved and prepared into dentine discs of varying thicknesses. Dentine discs that were 0.5 mm, 1.0 mm, and 1.5 mm thick were prepared and allocated into groups 1, 2, and 3, respectively.

The surfaces of the dentine discs representing the cavity side and the pulpal side of the remaining dentine were identified and marked. The surfaces of the dentine discs were ground using an EcoMet 5 wet grinder/polisher (Buehler) under running water to obtain the desired thickness and flatten the surface. A series of 300- to 2000-grit silicon carbide (SiC) papers (Buehler) was used. The discs were then treated in decreasing serial dilution of 17%, 10%, and 5% ethylenediaminetetraacetic acid to remove the smear layer and open the dentinal tubules.^21^

### Measurement of number and diameter of dentinal tubules of the dentine discs

Scanning electron microscopy (SEM) was used to examine the dentine discs. The dentine discs were mounted on aluminium stubs with the cavity side on top. The discs were then coated with a thin layer of gold/palladium using a Magnetron ion sputter metal coating device (MSP-2S, IXRF Systems, Inc., Austin, TX, USA). Following this, the samples were visualised with an SEM (SU-1500, Hitachi) at a consistent working distance of 8.6 mm and an accelerating voltage of 15 kV, which was chosen to optimise image quality.

For each dentine disc, 5 micrographs were captured from random fields at a fixed magnification of 3000×. The number of dentinal tubules present in each micrograph was recorded. Furthermore, the diameter of 5 randomly selected dentinal tubules from each micrograph was measured via SEM. Two perpendicular intersecting measurement lines were drawn to obtain the diameter of a dentinal tubule, and their mean value was calculated.

### Setup of experimental device

**Fig. 1:**
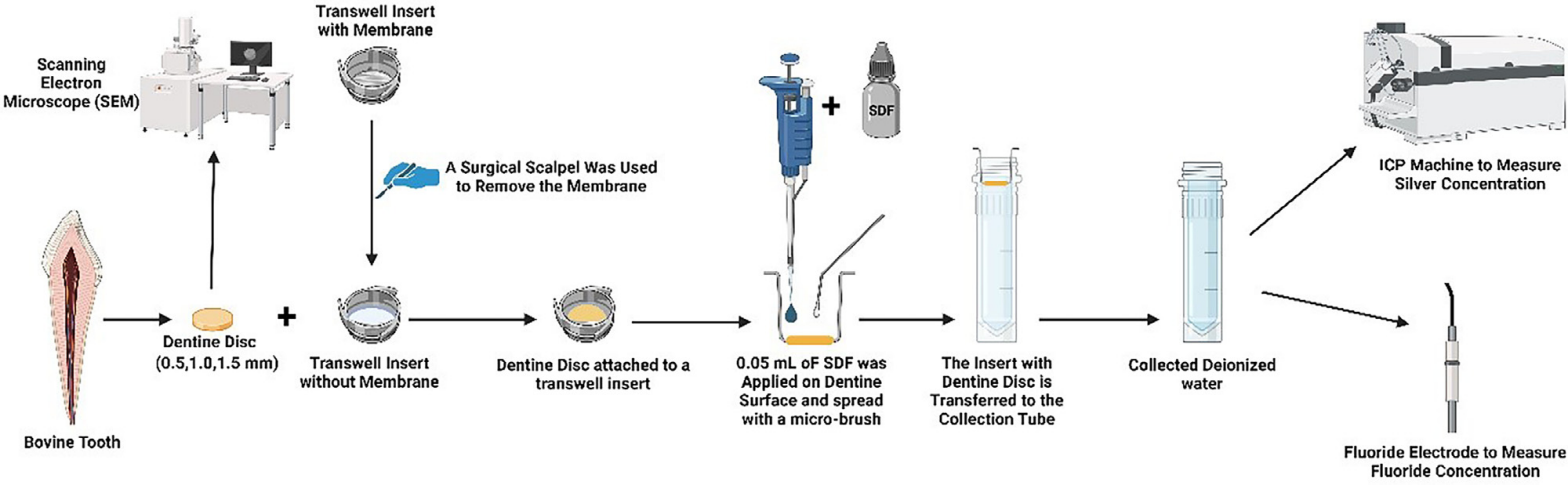
Flow diagram of the study. Figure was created with BioRender.com.

This device was set up to simulate the condition of ion diffusion into dental pulp through dentine following SDF application on the dentine of the cavity floor of the caries lesions. Transwell inserts of a 24-well plate (Corning Costar) were used to carry the dentine discs. The permeable membrane of the inserts was removed. The dentine discs were attached and fixed to the inserts using cyanoacrylate glue (Supa Glue, Selleys). Then, 38% SDF solution (Advantage Arrest, Oral Science) was applied to the cavity side of the dentine disc according to the manufacturer's instructions.^22^ Briefly, 0.05 mL of SDF was collected via pipette and applied on the cavity side of each dentine disc using a disposable micro-brush (3M ESPE) and left to soak for 1 minute (Figure 1).

Each dentine disc carried by the Transwell insert was transferred to a polystyrene container (Sterilin 7 mL Polystyrene Bijou Containers, Thermo Fisher Scientific). The tubes were filled with 5.8 mL of deionised water, so that the pulpal side of the dentine disc was submerged in the deionised water (Figure 1). The tubes with deionised water were collected on a weekly basis for 24 weeks. After each collection, a new tube filled with 5.8 mL of deionised water was used for subsequent ion collection to avoid contamination from any precipitating ions.

### Measurement of silver ion diffusion

A 2-mL amount of the collected solution of each sample was used for measurement of the silver ions, and then 2 mL of 2% nitric acid were added to each 2 mL of the collected solution to bring up the volume to 4 mL. The measurement of the free silver ion concentration was carried out using inductively coupled plasma–optical emission spectrometry (ICP-OES). A charge-coupled device detector of ICP-OES (Varian 720-ES) was used. ICP-OES was performed at 1 kW (RF power) with a plasma flow rate of 15 L/min. Briefly, a nebuliser converted the 4 mL of solution into a fine spray, and it was then mixed with argon in a spray chamber. The sample was subsequently added to plasma and instantly got excited by the high temperature, which allowed the silver content of the solution to be measured with an emission line of OD_328 nm_.

### Measurement of fluoride ion diffusion

In each well of a 24-well plate, 1 mL of each sample was added to 1 mL of TISAB II (total ionic strength adjustment buffer; Orion 940909, Thermo Fisher Scientific), which is a pH-adjusted (pH 6.0) buffer solution, and followed by thorough stirring using a magnetic stirrer. A fluoride ion–selective electrode (Orion 9609BNWP, Thermo Fisher Scientific) coupled with an Oakton Ion 2700 Benchtop Meter (Cole-Pamer) was used to measure the fluoride concentration. Calibration was done before measurement using 0.01-, 0.1-, 1-, 10-, and 100-ppm fluoride solutions prepared from an external fluoride standard of 0.1 M sodium fluoride (Orion 940907, Thermo Fisher Scientific). The electric potential of each solution was measured using the electrode. A linear regression against the standard fluoride concentrations (*R*^2^ > 0.99) was generated by plotting a line graph. The fluoride concentration was determined using the standard curve from the measured electric potential of the tested solution. Each measurement was repeated 3 times to obtain respective mean values for a set sample.

### Statistical analysis

The obtained data were entered in a MS Excel spreadsheet to obtain mean and SD for respective test groups. The data were then transferred to IBM SPSS Statistics v. 28.0.1.0 software. Multivariate analysis of variance (MANOVA) was used to determine the differences in number and diameter of dentinal tubules in dentine discs of varying thicknesses amongst the groups.

To explore the potential effects of the key factors in this study (thickness of dentine disc, number and diameter of dentinal tubules of varying dentine thickness, and time) on the obtained fluoride and silver concentrations. To analyse these relationships, the generalised estimating equations (GEE) method, specifically with an exchangeable correlation structure, was used to consider the intraclass correlation of the repeated measurements on the samples over time.

## Results

### Number and diameter of the dentinal tubules of the dentine discs

**Fig. 2:**
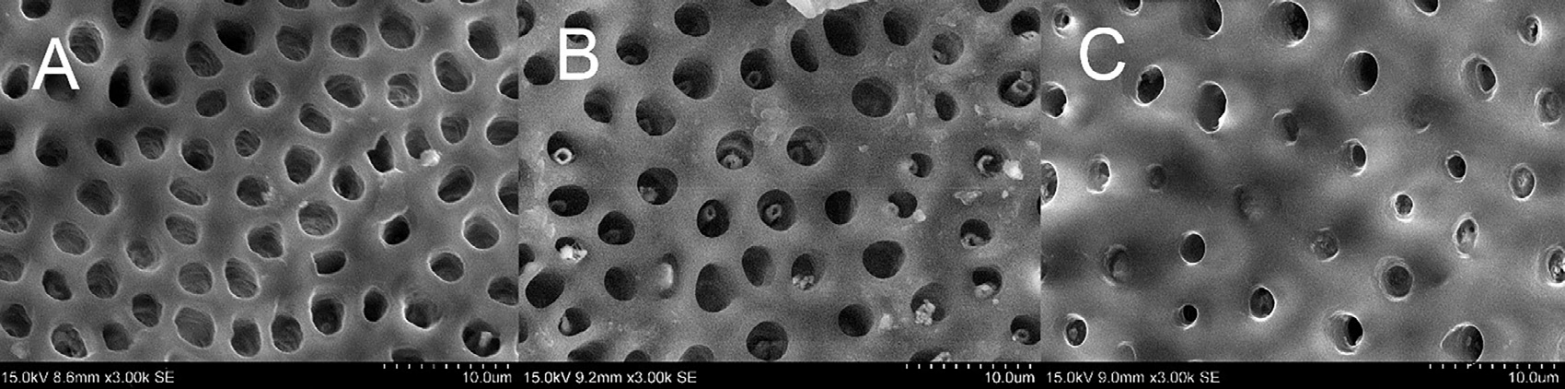
Scanning electron microscopy image the top (cavity) side of the dentine discs with different thicknesses. **A**, Dentine with 0.5 mm thickness. **B**, Dentine with 1.0 mm thickness. **C**, Dentine with 1.5 mm thickness.

The SEM images of the surfaces of varying dentine thicknesses are shown in Figure 2. The mean values of dentinal tubules’ diameters and numbers are displayed in Table 1. MANOVA results showed that there was a significant difference amongst the 3 groups in terms of dentinal tubules’ numbers and diameters (*P* < .05). The widest diameter and highest number of dentinal tubules were both recorded in group 1 (dentine thickness = 0.5 mm), whilst the lowest values were recorded in group 3 (dentine thickness = 1.5 mm).

**Table 1:** Values of dentinal tubules’ number and diameter in dentine discs with different thicknesses.

Parameter	Group 1 (0.5 mm), mean (SD)	Group 2 (1.0 mm), mean (SD)	Group 3 (1.5 mm), mean (SD)
Number	63 (2.9)	48.8 (0.8)	24.6 (3.2)
Diameter (µm)	3.4 (0.07)	2.9 (0.1)	2.1 (0.2)

#### Silver ion diffusion

**Fig. 3:**
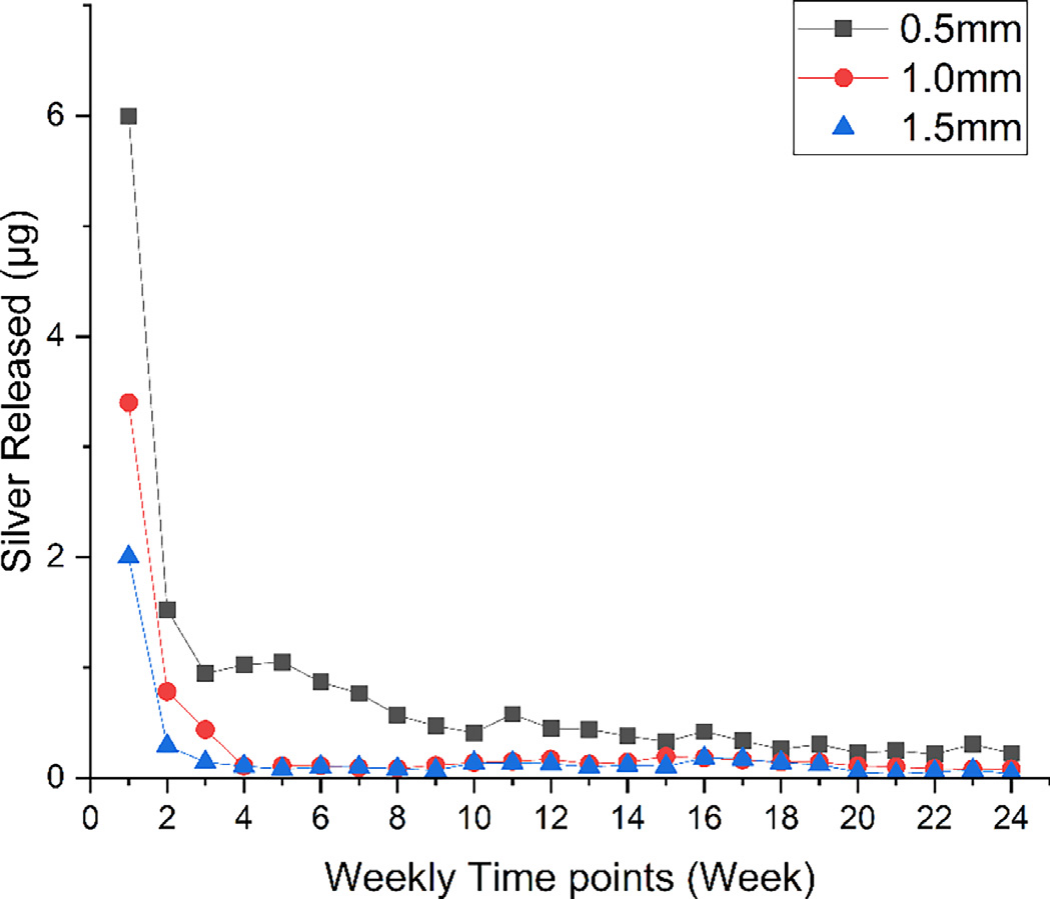
Trend of silver ion diffusion through dentine discs with different thicknesses after silver diamine fluoride solution application for 6 months.

The mean values of silver diffusion for each time interval are shown in Table 2 and Figure 3. A statistically significant difference in silver ion diffusion was found amongst all groups over the 6 months studied (*P* < .05). The highest mean value of silver ions was recorded at week 1 for all groups.

**Table 2:** Values of amounts of silver ion diffusion in different groups at different time points.^a^^,^^b^^,^^c^^,^^d^

Time point	Group 1 (dentine of 0.5 mm thickness), μg, mean (SD)	Group 2 (dentine of 1.0 mm thickness), μg, mean (SD)	Group 3 (dentine of 1.5 mm thickness), μg, mean (SD)
Week 1	6 (1)^A,1^	3.4 (0.7)^B,1^	2 (0.5)^B,1^
Week 2	1.5 (0.35)^A,2^	0.8 (0.2)^B,2^	0.3 (0.07)^C,2^
Week 3	0.9 (0.24)^A,3,4,5^	0.4 (0.1)^B,2,3^	0.1 (0.04)^C,2^
Week 4	1 (0.24)^A,3,4^	0.1 (0.2)^B,3^	0.1 (0.01)^B,2^
Week 5	1.1 (0.17)^A,3,4^	0.1 (0.02)^B,3^	0.1 (0.02)^B,2^
Week 6	0.9 (0.21)^A,3–5^	0.1 (0.02)^B,3^	0.1 (0.02)^B,2^
Week 7	0.8 (0.19)^A,3,5,6,9^	0.1 (0.02)^B,3^	0.1 (0.02)^B,2^
Week 8	0.6 (0.12)^A,5,6,9–11^	0.1 (0.02)^B,3^	0.1 (0.01)^B,2^
Week 9	0.5 (0.10)^A,6,7,9,10–12^	0.1 (0.03)^B,3^	0.1 (0.01)^B,2^
Week 10	0.4 (0.1)^A,6–15^	0.1 (0.04)^B,3^	0.1 (0.02)^B,2^
Week 11	0.6 (0.14)^A,5–7,9–11^	0.2 (0.03)^B,3^	0.1 (0.02)^B,2^
Week 12	0.4 (0.09)^A,6,7,9–13,15^	0.2 (0.04)^B,3^	0.1 (0.03)^B,2^
Week 13	0.4 (0.09)^A,6,7,9–15^	0.1 (0.01)^B,3^	0.1 (0.02)^B,2^
Week 14	0.4 (0.09)^A,6,7,9–15^	0.1 (0.02)^B,3^	0.1 (0.02)^B,2^
Week 15	0.3 (0.07)^A,7,10–15^	0.2 (0.04)^B,3^	0.1 (0.02)^B,2^
Week 16	0.4 (0.05)^A,6,7,9–15^	0.2 (0.04)^B,3^	0.2 (0.04)^B,2^
Week 17	0.3 (0.02)^A,7,10–15^	0.2 (0.03)^B,3^	0.2 (0.04)^B,2^
Week 18	0.3 (0.04)^A,7,10–15^	0.1 (0.04)^B,3^	0.1 (0.03)^B,2^
Week 19	0.3 (0.05)^A,7,10–15^	0.1 (0.02)^B,3^	0.1 (0.03)^B,2^
Week 20	0.2 (0.06)^A,8,10–15^	0.1 (0.01)^B,3^	0.1 (0.03)^A,B,2^
Week 21	0.2 (0.06)^A,7,10–15^	0.1 (0.02)^B,3^	0.1 (0.01)^B,2^
Week 22	0.2 (0.05)^A,8,11–15^	0.1 (0.01)^A,3^	0.1 (0.03)^A,2^
Week 23	0.3 (0.06)^A,7,10–15^	0.1 (0.01)^B,3^	0.2 (0.02)^B,2^
Week 24	0.2 (0.06)^A,8,10–15^	0.1 (0.03)^A,B,3^	0.1 (0.004)^B,2^
Cumulative value	20 (4)**	10 (2)*	5 (1)

The three groups differ significantly from each other, as indicated by the double asterisks used to mark that group one is different to both groups 2 and 3.

a One drop of silver diamine fluoride solution contains 4.74 mg of silver.

b The safety range of silver is 0.005 mg/kg/day^21^.

c Uppercase letters represent differences amongst groups at a single time point.

d Numbers 1 through 14 represent differences within the same group at different time points.

⁎ Significant difference (p<0.05).

At week 1, the highest mean (SD) value for silver diffusion at 6 (1) µg was recorded in group 1, followed by group 2 at 3 (0.7) and group 3 at 2 (0.5) µg, respectively. In group 1, there was a significant difference representing the decrease in the diffusion rate of silver from week 1 and at each time point up to week 4 (*P* < .05), followed by a much slower diffusion rate compared to the previous weeks. In group 2, there was a significant difference in silver diffusion rate at each time point from week 1 until week 3 (*P* < .05) and continued without any significant difference until the end of the study. In group 3, the highest diffusion rate was recorded at week 1 and was followed by a slow diffusion rate over the subsequent weeks. Cumulative mean values of silver ion diffusion in different groups are presented in Table 2.

#### Fluoride ion diffusion

**Fig. 4:**
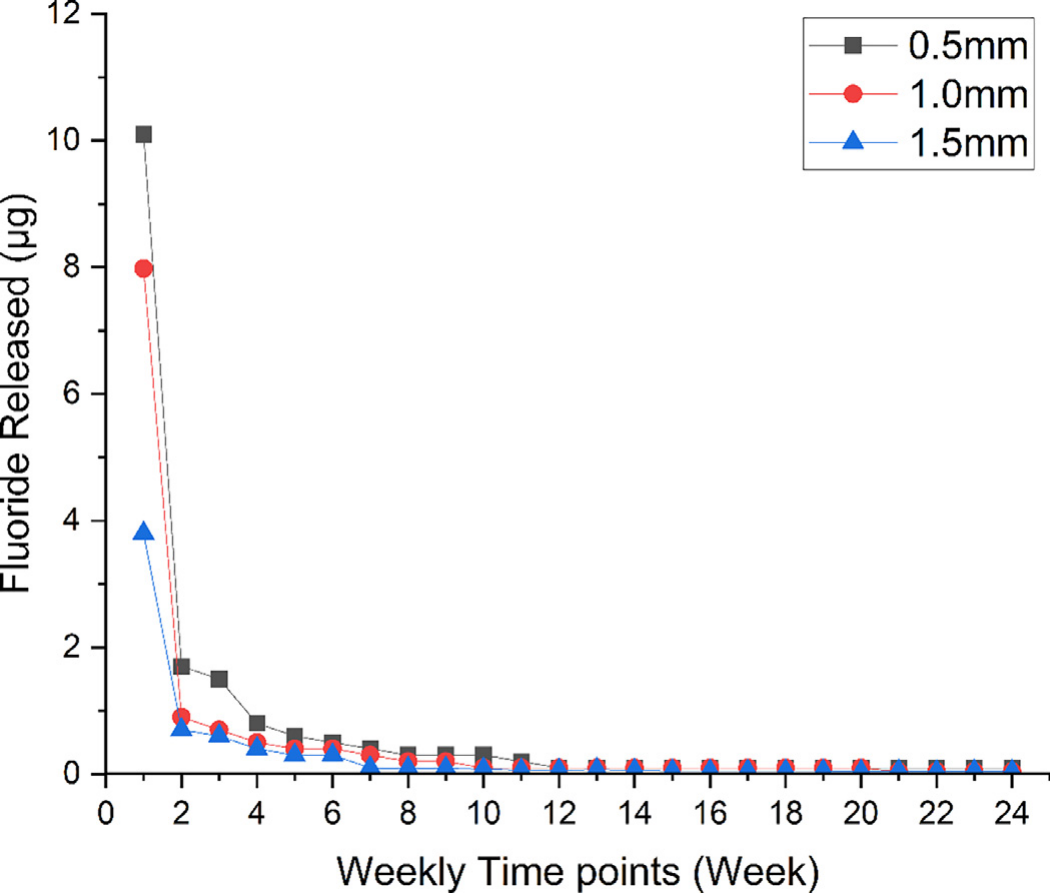
Trend of fluoride ion diffusion through dentine discs with different thicknesses after silver diamine fluoride solution application for 6 months.

The mean values of fluoride ion diffusion through dentine by week are shown in Table 3 and Figure 4. A statistically significant difference in fluoride ion diffusion was found amongst all groups over the 24 weeks of this study (*P* < .05). The mean (SD) maximum fluoride diffusion rate was recorded in group 1 at week 1 at 10.1 (0.19) µg, followed by group 2 at 7.98 (0.13) µg and group 3 at 3.8 (0.07) µg, respectively.

**Table 3:** Values of amounts of fluoride ion diffusion in different groups at different time points.^a^^,^^b^^,^^c^^,^^d^

Time point	Group 1 (dentine of 0.5 mm thickness), µg, mean (SD)	Group 2 (dentine of 1.0 mm thickness), µg, mean (SD)	Group 3 (dentine of 1.5 mm thickness), µg, mean (SD)
Week 1	10.1 (0.19)^X,1^	7.98 (0.13)^Y,1^	3.8 (0.07)^Z,1^
Week 2	1.7 (0.1)^X,2^	0.9 (0.1)^Y,2^	0.7 (0.09)^Z,2^
Week 3	1.5 (0.03)^X,3^	0.7 (0.1)^Y,3^	0.6 (0.01)^Z,3^
Week 4	0.8 (0.02)^X,4^	0.5 (0.01)^Y,4^	0.4 (0.01)^Z,4^
Week 5	0.6 (0.01)^X,5^	0.4 (0.01)^Y,5^	0.3 (0.01)^Z,4,5^
Week 6	0.5 (0.03)^X,6^	0.4 (0.04)^Y,5^	0.3 (0.01)^Z,5^
Week 7	0.4 (0.04)^X,7^	0.3 (0.03)^Y,6^	0.1 (0.004)^Z,6,8^
Week 8	0.3 (0.01)^X,8,10^	0.2 (0.03)^Y,7^	0.09 (0.004)^Z,6,7^
Week 9	0.3 (0.02)^X,8–11^	0.2 (0.01)^Y,7^	0.09 (0.003)^Z,6,7^
Week 10	0.3 (0.03)^X,8–11^	0.1 (0.01)^Y,7,8^	0.08 (0.005)^Z,6,7^
Week 11	0.2 (0.01)^X,9–11^	0.1 (0.01)^Y,8,9^	0.06 (0.004)^Y,6,7^
Week 12	0.1 (0.01)^X,12^	0.1 (0.003)^X,Y,8,9^	0.06 (0.0006)^Y,6,7^
Week 13	0.1 (0.01)^X,12^	0.1 (0.01)^X,8,9^	0.07 (0.003)^X,6,7^
Week 14	0.1 (0.002)^X,12^	0.1 (0.001)^X,Y,8,9^	0.06 (0.001)^Y,6,7^
Week 15	0.1 (0.004)^X,12^	0.1 (0.001)^X,Y,9^	0.05 (0.001)^Y,7^
Week 16	0.1 (0.003)^X,12^	0.1 (0.004)^X,Y,9^	0.05 (0.0004)^Y,7^
Week 17	0.1 (0.004)^X,12^	0.1 (0.002)^X,9^	0.05 (0.001)^X,7,8^
Week 18	0.1 (0.02)^X,12^	0.1 (0.003)^X,9^	0.05 (0.001)^X,7^
Week 19	0.1 (0.03)^X,12^	0.1 (0.004)^X,9^	0.05 (0.003)^X,7^
Week 20	0.1 (0.01)^X,12^	0.1 (0.002)^X,9^	0.04 (0.001)^X,7^
Week 21	0.1 (0.003)^X,12^	0.04 (0.001)^X,9^	0.05 (0.001)^X,7^
Week 22	0.1 (0.01)^X,12^	0.04 (0.001)^X,9^	0.04 (0.002)^X,7^
Week 23	0.1 (0.01)^X,12^	0.04 (0.002)^X,9^	0.05 (0.001)^X,7^
Week 24	0.1 (0.004)^X,12^	0.04 (0.002)^X,9^	0.04 (0.001)^X,7^
Cumulative value	18 (0.8)**	15 (0.6)*	7 (0.4)

The three groups differ significantly from each other, as indicated by the double asterisks used to mark that group one is different to both groups 2 and 3.

a One drop of silver diamine fluoride solution contains 2.24 mg of fluoride.

b The safety range of fluoride is 0.06 mg/kg/day^22^.

c Uppercase letters represent differences amongst groups at a single time point.

d Numbers 1 through 11 represent differences within the same group at different time points.

⁎ Significant difference (p<0.05).

In group 1, there was a significant decrease in the diffusion rate of fluoride from week 1 to week 11 (*P* < .05), followed by a much slower diffusion rate compared to the previous weeks. In group 2, there was a significant difference in fluoride diffusion rate at each time point from week 1 until week 10 (*P* < .05) and continued without any significant difference in fluoride ion diffusion until the end of the study. In group 3, there was a significant difference in fluoride diffusion rate at each time point from week 1 until week 5 (*P* < .05), followed by a low diffusion rate over the subsequent weeks.

#### Factors affecting silver and fluoride ion diffusion

Tables 4 and 5 display the outcomes of the GEE regression analysis for potential correlations within the data. The findings of this analysis revealed a notable influence of factors on the diffusion of fluoride and silver ions through dentine. Specifically, our results demonstrated that a reduction in dentine thickness led to a higher rate of ion diffusion, which can be attributed to the concomitant increase in both the number and diameter of the dentinal tubules.

**Table 4:** Estimated coefficients and significance of predictor variables from the generalised estimating equations model for silver ion diffusion.

Parameter	β	95% Wald CI		Significance
		Lower	Upper	
[Group=1] * Time point in weeks	16.171	11.194	21.147	<.001
[Group=2] * Time point in weeks	−118.728	−156.882	−80.573	<.001
[Group=3] * Time point in weeks	0^a^	.	.	.
[Group=1] * Tubule diameter	82.178	60.172	104.184	<.001
[Group=2] * Tubule diameter	−611.823	−836.627	−387.018	<.001
[Group=3] * Tubule diameter	0^a^	.	.	.
[Group=1] * Number of tubules	4.351	3.171	5.531	<.001
[Group=2] * Number of tubules	−35.860	−48.993	−22.728	<.001
[Group=3] * Number of tubules	0^a^	.	.	.
[Group=1] * Number of tubules * Tubule diameter	−1.303	−1.658	−0.949	<.001
[Group=2] * Number of tubules * Tubule diameter	12.794	8.088	17.499	<.001
[Group=3] * Number of tubules * Tubule diameter	0^a^	.	.	.
[Group=1] * Number of tubules * Time point in weeks	−0.258	−0.338	−0.178	<.001
[Group=2] * Number of tubules * Time point in weeks	2.483	1.684	3.282	<.001
[Group=3] * Number of tubules * Time point in weeks	0^a^	.	.	.
[Group=1] * Tubule diameter * Time point in weeks	−4.881	−6.373	−3.390	<.001
[Group=2] * Tubule diameter * Time point in weeks	42.337	28.658	56.015	<.001
[Group=3] * Tubule diameter * Time point in weeks	0^a^	.	.	.
[Group=1] * Number of tubules * Tubule diameter * Time point in weeks	0.077	0.053	0.101	<.001
[Group=2] * Number of tubules * Tubule diameter * Time point in weeks	−0.885	−1.172	−0.599	<.001
[Group=3] * Number of tubules * Tubule diameter * Time point in weeks	0^a^	.	.	.

⁎ β coefficient is known as a partial regression coefficient.

a This denotes that Group 3 is the baseline or reference group in this analysis

**Table 5:** Estimated coefficients and significance of predictor variables from the generalised estimating equations model for fluoride ion diffusion.

Parameter	β	95% Wald CI		Significance
		Lower	Upper	
[Group=1] * Time Point in weeks	1.352	0.665	2.040	<.001
[Group=2] * Time Point in weeks	4.014	2.974	5.053	<.001
[Group=3] * Time Point in weeks	0^a^	.	.	.
[Group=1] * Tubule diameter	5.754	2.117	9.392	.002
[Group=2] * Tubule diameter	23.852	19.371	28.334	<.001
[Group=3] * Tubule diameter	0^a^	.	.	.
[Group=1] * Number of Tubules	0.304	0.109	0.499	.002
[Group=2] * Number of Tubules	1.380	1.118	1.642	<.001
[Group=3] * Number of Tubules	0^a^	.	.	.
[Group=1] * Time Point in weeks * Tubule diameter	−0.410	−0.616	−0.204	<.001
[Group=2] * Time Point in weeks * Tubule diameter	−1.460	−1.832	−1.088	<.001
[Group=3] * Time Point in weeks * Tubule diameter	0^a^	.	.	.
[Group=1] * Time Point in weeks * Number of Tubules	−0.022	−0.033	−0.011	<.001
[Group=2] * Time Point in weeks * Number of Tubules	−0.085	−0.106	−0.063	<.001
[Group=3] * Time Point in weeks * Number of Tubules	0^a^	.	.	.
[Group=1] * Diameter * Number of Tubules	−0.087	−0.145	−0.028	.004
[Group=2] * Diameter * Number of Tubules	−0.498	−0.591	−0.404	<.001
[Group=3] * Diameter * Number of Tubules	0^a^	.	.	.
[Group=1] * Time Point in weeks * Tubule diameter * Number of Tubules	0.006	0.003	0.010	<.001
[Group=2] * Time Point in weeks * Tubule diameter * Number of Tubules	0.030	0.023	0.038	<.001
[Group=3] * Time Point in weeks * Tubule diameter * Number of Tubules	0^a^	.	.	.

⁎ β coefficient is known as a partial regression coefficient.

a This denotes that Group 3 is the baseline or reference group in this analysis

## Discussion

To the authors’ knowledge, this is the first study to quantify the amount of silver and fluoride ions diffusing across varying dentine thicknesses after the topical application of 38% SDF over 24 weeks. We also explored the impact of thickness of dentine and dentinal tubules’ diameter and number on the diffusion of silver and fluoride ions in dentine. The null hypothesis was rejected due to the identification of significant differences in the mean values of silver and fluoride diffusion across all groups.

Throughout this study, recorded silver ion levels passing through dentine of varying thicknesses remained at a very low level. The potential health risks due to silver absorption are considered minimal, as highlighted by Lansdown.^23^ Duangthip et al^24^ reported that the silver content in a treatment involving 3 teeth is approximately 7.6 mg of SDF, translating to about 1.50 mg of silver for the 3 teeth, or 0.5 mg per tooth. The results of our study indicated that the rate of silver diffusion potentially reaching the dental pulp was approximately 0.02 mg silver (4% of the applied silver ions). Over the 6 months of the study, the amount of silver reaching the pulp was much lower than the safe threshold for oral exposure and decreased with increasing dentine thickness.^25^

The levels recorded for fluoride ions penetrating through dentine discs of varying thicknesses were very low and within safe thresholds for oral exposure.^26^^,^^27^ According to Vasquez et al,^28^ a single application of 38% SDF typically contains 0.33 mg of fluoride. The threshold for fluoride toxicity is generally considered to be around 5 mg/kg.^29^ Based on the results of our study, in the case of 0.5 mm of remaining dentine thickness, approximately 0.05% of the applied fluoride dose reaches the dental pulp and the amount of fluoride will decrease with increasing dentine thickness.

The decrease in the diffusion rate of fluoride ions happened much more quickly compared to silver ions. This might be attributed to the difference in size between the 2 ions. The atomic radius of silver is 144 pm, which is double that of fluoride, at 72 pm. This difference in size might be the reason for the difference in diffusion rate of the 2 ions.

The penetrating amount of both silver and fluoride ions was significantly different amongst different thicknesses of dentine discs following SDF application in the current study. It is easier for ions to travel with the reduced amount of dentine acting as a barrier. The results prove that the thickness of dentine plays a pivotal role in influencing the rate and quantity of substances diffusing through it, which is consistent with the findings of several previous studies. Previous studies have shown that as the thickness of remaining dentine decreased, there was an increase in the permeability or leaching of substances through the dentine barrier.^30^^,^^31^

The thickness of the remaining dentine reflects the properties of the dentinal tubules at different locations. The dentinal tubules in the circumferential pulpal dentine are greater in number and wider in diameter, as seen in SEM images.^32^ This study proved that there was an inversely proportional relation between dentine thickness and the amount of silver and fluoride ions that could pass through it. Therefore, the capacity of remaining dentine regulating the quantity of silver and fluoride ions reaching the dental pulp was correlated to the remaining dentine thickness and the number and diameters of the dentinal tubules in the cavity floor; this was proved statistically as displayed in the results section. Although the diameter and the density of the dentinal tubules significantly affect the rate of ion diffusion, it is difficult to measure these 2 parameters in clinical settings. The thickness of the remaining dentine is more convenient to assess clinically. Therefore, extra care should be taken when evaluating the thickness of remaining dentine during clinical work.

The GEE model was used to analyse the correlation of ion releasing and the intrinsic properties of dentine specimen. The exchangeable correlation structure chosen for the GEE test assumes that any 2 observations within the same cluster (in this case, measurements from the same sample) are equally correlated. This was based on our assumption that the correlation between any 2 measurements taken from the same sample would be approximately the same, irrespective of the time points at which these measurements were taken. In addition, the intraclass correlation of the repeated measurements on the samples over time was taken into account. Intraclass correlation reflects the degree of similarity of the repeated measurements from the same sample. It is crucial to consider this in our analysis, as ignoring it could lead to incorrect conclusions about the effects of our factors of interest. Considering intraclass correlation leads to the acknowledgement that the repeated measurements are not entirely independent but are grouped by each individual sample. In this way, by using the GEE model with an exchangeable correlation structure and considering the intraclass correlation of our repeated measurements, we were able to effectively explore the effects of dentine thickness, the number and diameter of dentinal tubules, and time on the concentrations of silver and fluoride ions obtained from the dentinal tubules.

This study spanned 6 months, and the readings were collected weekly for 24 weeks. This time frame aligns with the recommended biannual application of SDF for enduring effects.^33^ In a study by Zhi et al,^34^ the frequency of SDF application was found to have a significant effect on carious dentine hardening. The biannual application increased the portion of the active dentinal lesion that became arrested. Hence, the 24-week period was selected for this study.

This study provided promising results for the application of SDF as an indirect pulp-capping agent. The anticariogenic effect of SDF has been well established through multiple studies.^6^^,^^7^^,^^35^ SDF aids in the remineralisation and hardening of demineralised dentine, and it possesses antibacterial properties.^36^ In a systematic review published by our group discussing the effect of SDF application as an indirect capping agent, the results showed an enhancement of the dentine–pulp complex repair process.^16^ This enhancement was manifested as formation of tertiary dentine, presence of normal pulp architecture, only mild inflammation, and absence of silver particles inside the pulp. A randomised controlled trial (RCT) by Baraka et al^37^ used 3-dimensional radiographic assessment to confirm the formation of tertiary dentine when SDF was used as an indirect capping agent. In various RCTs, SDF was compared to calcium hydroxide (CaOH) as indirect capping agents.^38^, ^39^, ^40^, ^41^ These studies revealed that there was no significant difference in the outcomes between the 2 materials. However, SDF holds an advantage over CaOH due to its ease of handling and application. Furthermore, SDF does not impact the mechanical characteristics of the restorative material applied over it, unlike CaOH, which is recognised for its inferior mechanical properties.^42^

SDF serves as a mechanical barrier against carious stimuli, and it triggers an adaptive pulpal response that promotes enhanced mineral deposition by odontoblasts.^43^ This response acts as a protective defence mechanism against microbial stimuli. The findings of this study indicate that SDF can be safely employed as a cavity liner without risking toxicity to the dental pulp. When SDF is combined with KI, the diffusion rate is decreased.^17^ Its application on the dentine of older individuals, where permeability is significantly reduced, can also be beneficial, especially in scenarios with a high likelihood of recurrence.

Limitations of this study include the absence of intrapulpal pressure, which might substantially reduce the diffusion rate.^44^ In addition, in this study only the diffusion rate of silver and fluoride ions was assessed, but not the effect of these ions on the dental pulp cells. Further in vivo and clinical studies on the effect of SDF on pulp cells are warranted.

As this research was conducted amidst the COVID-19 pandemic, the challenge of acquiring human teeth specimens necessitated the use of bovine teeth as a substitute. Dentine discs were cut and taken from the region adjacent to the cementoenamel junction for our experiments. Schmalz et al^45^ identified that dentine from this particular area is a suitable alternative for human coronal dentine in vitro, particularly in studies assessing transdentinal permeability. Moreover, Schilke et al^46^ demonstrated that there are no significant differences in the number or diameter of dentinal tubules per square millimeter between human and bovine dentine.

## Conclusions

Silver and fluoride ion diffusion through dentine showed a decline trend over 24 weeks following SDF application. The thickness of the dentine, which affects the diameter of the dentinal tubules and the number of dentinal tubules, plays a significant role in determining the diffusion process of silver and fluoride ions from SDF through the dentine.

## Conflict of interest

The authors state that they have no conflict of interest.
